# Effects of Microwaves, Ultrasonication, and Thermosonication on the Secondary Structure and Digestibility of Bovine Milk Protein

**DOI:** 10.3390/foods11020138

**Published:** 2022-01-06

**Authors:** Jin Wang, Rachit Saxena, Sai Kranthi Vanga, Vijaya Raghavan

**Affiliations:** 1 Key Laboratory of Environmental Medicine and Engineering, Ministry of Education, Department of Nutrition and Food Hygiene, School of Public Health, Southeast University, Nanjing 210009, China; 2 Department of Bioresource Engineering, Faculty of Agricultural and Environmental Sciences, McGill University, 21111 Lakeshore Rd, Sainte-Anne-de-Bellevue, QC H9X 3V9, Canada; rachit.saxena@mail.mcgill.ca (R.S.); sai.vanga@mail.mcgill.ca (S.K.V.); vijaya.raghavan@mcgill.ca (V.R.)

**Keywords:** cow’s milk, protein secondary structure, beneficial ultrasound treatment, thermosonication treatment, protein digestibility

## Abstract

Cow’s milk is considered an excellent protein source. However, the digestibility of milk proteins needs to be improved. This study aimed to evaluate the relationship between the functional properties of milk proteins and their structure upon microwave, ultrasound, and thermosonication treatments. The protein content, digestibility, and secondary-structure changes of milk proteins were determined. The results demonstrated that almost 35% of the proteins in the untreated samples had a α-helix structure and approximately 29% a β-sheet and turns structure. Regarding the untreated samples, the three treatments increased the α-helices and correspondingly decreased the β-sheets and turns. Moreover, the highest milk protein digestibility was observed for the ultrasound-treated samples (90.20–94.41%), followed by the microwave-treated samples (72.56–93.4%), whereas thermosonication resulted in a lower digestibility (68.76–78.81%). The milk protein content was reduced as the microwave processing time and the temperature increased. The final milk protein available in the sample was lower when microwave processing was conducted at 75 °C and 90 °C compared to 60 °C, whereas the ultrasound treatment significantly improved the protein content, and no particular trend was observed for the thermosonicated samples. Thus, ultrasound processing shows a potential application in improving the protein quality of cow’s milk.

## 1. Introduction

Cow’s milk is considered an excellent food source for human body growth due its high content of minerals (calcium and phosphorous) and protein. Cow’s milk can provide all essential amino acids including a high level of lysine, which can help in synthesizing important proteins important for human health [1,2]. Milk proteins perform several functions such as immune system stimulation, shielding the human body against different types of bacteria, viruses, and fungi, and gut development [3,4]. Overall, milk is also found to have most of the required macro- and micronutrients to provide balanced nutrition, especially to children and infants [5]. However, the protein quality of cow’s milk still nees to be improved through various food-processing techniques due to these proteins’ low digestibility and their allergenicity, which could lead to gastrointestinal discomfort, respiratory failure, as well as anaphylactic shock [6].

Thermal processing of milk aids in the extension of its shelf life and in the reduction of microbial activity [7]. However, thermal process is known to induce some structural changes in milk, such as protein denaturation. It can further cause the permanent unfolding of protein and even might expose hydrophobic groups and reduce disulphide bridges [8,9]. A study reported that a microwave treatment caused a decrease in the content of lactose, fat, and protein in cow’s milk, whereas milk’s average density was increased [10]. There was a decrease in α-helixes and β-sheets when milk was treated with microwaves above 50 °C [11]. In recent years, non-thermal processing has received high attention because it can retain the original characteristics, freshness, and nutritional value compared to thermal treatment [7]. Studies reported that ultrasounds had a minor effect on the secondary structure and hydrophobicity of a whey protein concentrate [12]. In sodium caseinate (biochemical name of casein protein), study also concluded that during ultrasound treatment, there were no major structural deviations, but a slight deviation was observed for lactoferrin [13]. Moreover, during ultrasonication, there was minimal loss in flavor, and this processing technique exhibited higher consistency in terms of homogenization and viscosity compared to other non-thermal techniques [14]. In addition, ultrasound processing has a lower operating cost and an effective power output [15,16].

However, very limited studies regarding the combination of ultrasound and thermal treatments have been performed to improve the protein quality of cow’s milk. In the present study, microwaves, ultrasonication, and thermosonication treatments were conducted to study the relationship between the functional properties of milk proteins (protein and in vitro digestibility) and their secondary structure. Nowadays, many food industries and researchers across the globe are adopting non-thermal technologies for processing food products. Hence, it will be of great importance to compare the effect of thermal and non-thermal techniques on the protein structure and digestibility of cow’s milk proteins after processing.

## 2. Material and Methods

Raw cow’s milk was collected from the Macdonald dairy farm, McGill University (Lakeshore Ste Anne de Bellevue, Quebec, Canada), and was stored at refrigerated conditions (4 °C) until further processing. All samples were processed within 48 h from collection from the farm. All chemicals were bought from Sigma-Aldrich, Oakville, ON, Canada.

### 2.1. Microwave Treatment

Microwave processing of cow milk was conducted in a Mini WAVE Digestion Module (SCP Science, Montreal, Canada) that operates at a frequency of 2.45 GHz. Cow milk (30 mL) was transferred into a cylindrical quartz reactor vessel (100 mL), and then the milk was processed inside the Mini WAVE, which included 6 chambers designed to accompany the vessel. According to preliminary studies (50–100 °C, 0–10 min), the Mini WAVE was operated at 60, 75, and 90 °C for 1, 3, and 5 min, respectively (Table 1a). The processing time and temperature of the samples were monitored by Infrared Radiation (IR) sensors located at the bottom wall of the treatment chambers. The samples were then freeze-dried (LyoLab 3000, Thermo Scientific, Montreal, Canada) and stored at 4 °C until further analysis. All treatments were carried out in three replicates.

### 2.2. Ultrasound Treatment

The samples were obtained by transferring raw cow milk (30 mL) into Falcon tubes (50 mL), and ultrasound treatment was carried out using an ultrasound equipment (25 kHz and 400 W, Branson Ultrasonic Corp., Danbury, CT, USA). The ultrasonication (probe diameter, 12.7 mm) treatment was carried out flowing the method described by the previous study with slight modifications [17]. The treatment was carried out at 1, 3, 5, 7, and 9 min (Table 1b). An ice bath was used to avoid temperature changes during ultrasound processing. All the experiments were carried out in triplicates. All samples were freeze-dried after processing and stored at 4 °C until further analysis.

### 2.3. Thermosonication Treatment

According to the method described by the previous study, 30 mL of raw milk was transferred to 50 mL Falcon tubes and preheated in a water bath at 90 °C [18]. The Falcon tubes with the samples were taken out of the water bath when the temperature of milk reached 63 °C. The temperature changes were determined using a handheld thermometer. Then, the heated samples were immediately sonicated with a Branson Sonifier (25 kHz and 400 W, Branson Ultrasonic Corp., Danbury, CT, USA) for 1, 3, 5, 7, and 9 min (Table 1c). The samples were cooled down to room temperature, kept in the freezer, and freeze-dried for further analysis. All the experiments were carried out in triplicates.

### 2.4. Fourier-Transform Infrared Spectroscopy (FTIR)

The FTIR analysis was carried out by a Nicolet iS5 attenuated total reflectance (ATR)-FTIR spectrometer. The sample in powdered form (1 g) after freeze-drying were kept on the diamond crystal, and a clamp was used to tighten it for IR analysis. An average of 32 spectra at 4 cm^−1^ were taken for all samples (microwave, ultrasound, and thermosonication) to obtain the final spectrogram. For the background reference, the spectrum of an empty ATR diamond was obtained. The analysis of the raw data was conducted with OMNIC software (Version 8, Thermo Nicolet Instrument Corp., Madison, WI, USA), and Origin Pro (version 9, Origin lab corporation, Northampton, MA, USA) was used to obtain the area percentages of each secondary structure (beta sheets, alpha helix, beta turns, and random coils) according to the Amide I frequency range (Table 2). The spectra of treated and untreated sample were compared. All measurement were performed in triplicate.

### 2.5. Total Soluble Protein Determination

Two grams of freeze-dried milk samples was extracted with double-distilled water for 30 min at room temperature, and then the mixture was centrifuged at 5000× *g* for 10 min, and the supernatant was collected for further analysis. The total soluble protein content was determined with the Pierce BCA (bicinchoninic acid) protein assay kit, and the microplate procedure was adopted to analyze the protein content in the samples according to the instructions provided in the kit manual. The absorbance changes were recorded at 562 nm, and bovine serum albumin was used to obtain a standard curve. The protein content is expressed in g/100 g of dry samples.

### 2.6. In Vitro Enzymatic Protein Digestion (IVPD)

The in vitro enzymatic protein digestion was carried out with pepsin (≥2000 units/mg protein) and pancreatic (≥250 units/mg protein) enzymes [19,20]. The processed cow milk samples were freeze-dried, and 4 mg milk powder was dissolved in 1 mL of 0.01 M phosphate buffer. The pH of the solution was adjusted to 1.5 with the help of 1 N HCl. A pepsin enzyme solution (5 mg pepsin enzyme/mL in 0.01 N HCl) was then added, so that the enzyme-to-substrate ratio was maintained at 1:100 (*v*/*v*). The solution was maintained at 37 °C in a water bath with continuous stirring for 30 min, after which the digestion was interrupted by the addition of 1 M NaOH solution which raised the pH to 7.8. A pancreatic solution (5 mg pancreatic enzyme/mL in 0.1 M phosphate buffer) was added to the previously digested pepsin solution, so that the enzyme-to-substrate ratio was 1:30 (*v*/*v*). The samples were kept in a hot water bath, and the temperature was maintained at 40 °C. The digestion was stopped after 60 min with the addition of 150 mM Na_2_CO_3_. The protein content before and after digestion were determined using the Pierce BCA protein assay kit. The following equation was used to determine the in vitro protein digestion percentage [20]:

$$\mathrm{IVPD}\;\left(\%\right)=\frac{P0-P1}{P0\;}\times 100\;\left(1\right)$$

where P0 is the protein content before digestion, and P1 is the protein content after digestion.

### 2.7. Statistical Analysis

A one-way analysis of variance (ANOVA) was used to analyze the data obtained from each treatment. The significance was observed at *p* < 0.05 by Tukey’s test with the SPSS software. All treatments and measurements were carried out in three replicates.

## 3. Results and Discussion

### 3.1. FTIR Analysis

FTIR spectroscopy was used to investigate the structural changes in protein due to the processing techniques. Among all regions, the Amide I band ranging from 1700 to 1600 cm^−1^ was chosen for the study, as it is considered a sensitive region for the study of conformational changes occurring in a protein secondary structure. The Amide I band consist of C=O stretching vibrations (almost 80%) in addition to C–H stretching modes and in-plane N–H bending. The C=O stretching vibrations are the result of changes in the secondary structure of the protein as well as of inter- or intramolecular effects. The hydrogen bonding pattern and geometry of a molecule are also sometimes responsible for these vibrations [21,22]. The Amide I band consists of various secondary structural components such as β sheets, random coils, α helices, and β turns [9]. Table 2 shows various frequencies assigned to secondary structure components in the Amide I region.

**Figure 1:**
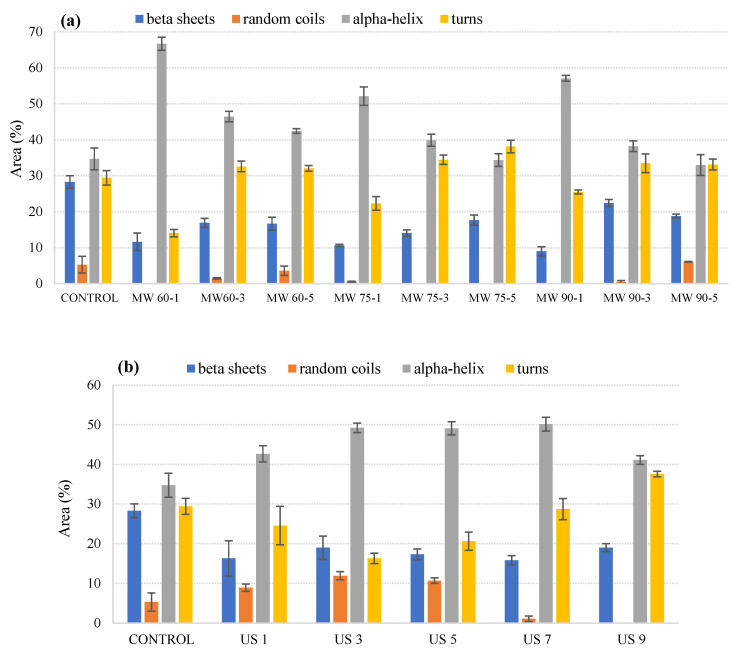

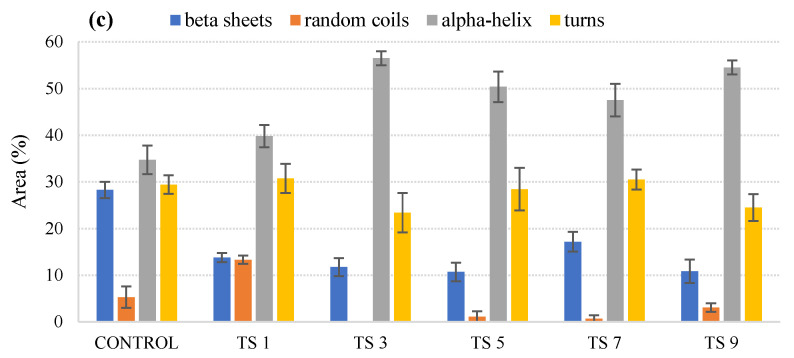
Variations in relative areas of the bands fitted to the normalized FTIR spectra of the Amide I region (1700–1600 cm^–^^1^) of microwave-processed (**a**), ultrasound-processed (**b**), and thermosonication-processed (**c**) cow milk. Note: the error bar means standard error of the mean.

We observed high peaks at 1624–1639 (β-sheets), 1642–1645 (random coils), 1648–1660 (α helix), and 1662–1697 (turns). The bands at 1611 cm^−1^ were not considered in this study, as their absorbance generally began at 1595 cm^−1^ and hence was not part of the Amide I region. The FTIR analysis was conducted by using OriginPro (Version 9, Origin Lab Corporation, Northampton, MA, USA). Figure 1a shows the percentage peak areas obtained for microwave-processed samples at 60 °C, 75 °C, and 90 °C for 1, 3, and 5 min, respectively, within the Amide I region. Figure 1b,c shows the peak areas obtained for milk processed with ultrasonication and thermosonication for 1, 3, 5, 7, and 9 min, respectively.

The control showed the presence of α-helices, β-sheets, and turns, each for about 30%. It was observed that approximately 50–60% of the secondary structure was composed of α helices during microwave processing at 60 °C, with a reduction in turns and β-sheets. There was a decrease in the α helix structure and an increase in turns at 60 °C as the processing time was increased from 1 to 5 min. The same trend was observed at 75 °C and 90 °C when the processing time was increased from 1 to 5 min, except for some slight deviations with a significant increase in random coils in MW 90-5 samples. In all the three temperature–time combinations, the reported β-sheets were lower than in the control. However, within each temperature set, they showed an increasing trend with an increase in the processing time, except for MW 90-5 for which random coils were observed. During all treatments, the random coil structure accounted for only 5% of the secondary structure. It can be inferred from Figure 1a that for each individual sets of temperature, as the treatment time progressed, an increase in random coil structure was observed at 60 °C and 90 °C, while the random coil was hardly found at 75 °C.

During ultrasound processing, no trend was observed. The α helix structure increased from 42.65% to 49.21% along with a decrease in turns from 24.56% to 16.30% as the treatment time increased from 1 to 3 min. There was also a slight increase in β-sheets and random coils during this time interval. When the treatment time was further increased to 5 min, turns showed a positive increment of 4.31%, with some slight deviations in α-helices, β-sheets, and random coils. There was a relocation of random coils as the area decreased from 10.71% to 1.09% when the processing time was increased to 7 min, with an increase in the area of turns as well as some variations in β-sheets and α helix, as shown in Figure 1b. There was again relocation of the α helix structure and an increase in β-sheets and turns as the processing time increased to 9 min. Overall, ultrasonication resulted in increased α helices in all ultrasonicated samples compared to the control.

The secondary structure of cow milk treated with combined thermal treatment and ultrasound waves showed a dominance of α helices and turns throughout the treatment time. There was a significant increase in the α helix structure (almost 15%) with a significant decrease in the area of turns and random coils when cow milk was processed for 3 min as compared to 1 min, as shown in Figure 1c. With a further increase in the treatment time from 3 min to 5 min and 7 min, there was a decrease in the α helix structure, with an increase in the area of turns. The β-sheets showed an irregular trend, as their area first decreased from 11.78% to 10.71% (from 3 to 5 min) and then again increased to 17.2% (from 5 to 7 min). There was a relocation of β-sheets and turns as the area of both structures showed a downward trend when the cow milk was processed for 9 min. Due to the relocation of both structures, an increase in the area of α helices was observed (approximately 7%). During the whole treatment, a non-significant presence of random coils was observed.

The results for thermal treatment showed a decrease in the α helix structure for all temperature–time combinations and, due to the relocation of the α helix structure, there was an increase in β-sheets and turns. These trends were also reported in previous studies [26,27] and suggested that the reduction in α helices exposed the free thiol group, Cys-121; this group causes aggregation of whey protein, which in turn increases the viscosity of milk. The increase in turns can contribute to the increased involvement of casein in forming aggregates, whereas the increase in β-sheets can be due to interactions between proteins and lipids, which tend to change the physical state of milk fat when stored at high temperatures [23,28]. Therefore, further research needs to be focused on the effect of ultrasound and thermosonication on protein structure and allergenicity, as deviations in protein structural affect functionality and hence allergenicity. Moreover, more stable α helix structures were found in ultrasonicated and thermo-sonicated samples, suggesting that non-thermal processing techniques do not affect the stability of proteins’ secondary structure.

### 3.2. Effect of Processing on Protein Digestibility

**Figure 2:**
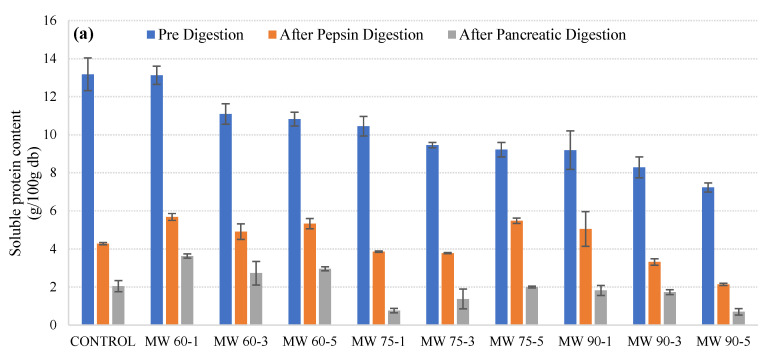

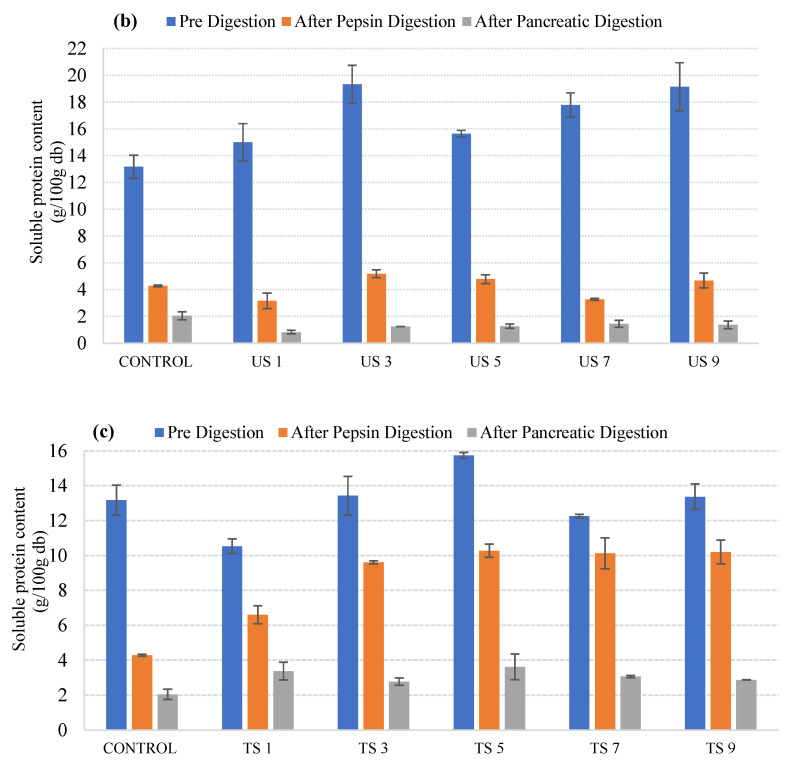
Protein content changes of microwave-processed (**a**), ultrasound-processed (**b**), and thermosonication-processed (**c**) cow milk under different conditions (pre-digestion, after pepsin digestion, and after pancreatic digestion).

In vitro digestion was carried out with pepsin and pancreatic enzymes in microwave-treated, ultrasound-treated, and thermosonicated samples. Figure 2a–c represents the soluble protein content of initial, intermediary, i.e., after pepsin digestion, and final, i.e., after pancreatic digestion, samples treated with microwaves, ultrasound waves, and a combination of thermal treatment and ultrasound waves. In Figure 2a, the initial soluble protein content in microwave-treated samples at 60 °C for 1, 3, and 5 min was significantly higher compared to the initial protein content at 75 °C and 90 °C. This could be because at higher temperatures, protein denatures, leading to the release of hydrophobic cores that expose hydrophobic residues to the surroundings. Furthermore, no trend was observed in protein content determined after pepsin digestion, but protein content was reduced after pancreatic digestion at 60 °C and 90 °C (except for some variations) and increased at 75 °C.

In Figure 2b, it can be observed that the initial soluble protein content was higher when the samples were treated with ultrasound compared to untreated samples. The increase in the soluble protein content could result from cavitation effects which lead to the transformation between soluble and insoluble proteins during ultrasound processing. The protein content after pepsin digestion showed an increase when the treatment time was increased from 1 min to 3 min, after which there was a slight decrease when the treatment time was raised to 5 min. The protein content after pepsin digestion again showed a raise as the treatment time increased to 7 min and 9 min. The final protein content after pancreatic protein digestion showed a different trend. The protein content values gradually increased from 1 min to 9 min, with the higher peak observed at 7 min. Figure 2c shows the pre-digestion, after-pepsin-digestion, and after-pancreatic-digestion protein content of the thermosonicated samples. It can be observed that the initial protein content increased after treatment for 1 min to 5 min, after which it showed a decrease at 7 min and a gradual increase at 9 min. The protein content after pepsin digestion showed an increasing trend, with slight variations when the treatment time was increased from 1 to 9 min. Furthermore, no particular trend was observed in protein content after pancreatic digestion for all treated samples.

Table 3a–c shows the IVPD (%) for microwave-processed, ultrasound-processed, and thermosonicated samples. During the thermal treatment, unfolding of tertiary and secondary structures (protein denaturation) occurs, and this phenomenon generally has a positive effect on protein digestibility [29]. Non-enzymatic post-transitional changes do occur in milk and negatively affect its digestibility. The Maillard reaction has the tendency to change the configuration of the side chain of proteins, particularly the ε-amino groups of Lysine [30]. Digestion by trypsin and other enzymes is hindered when there is a blockage in the ε-amino groups of Lysine residues during milk processing, which results in a reduction of protein digestibility [31,32,33]. There are other reactions such as β-elimination which involve cysteine/cystine (Cys) and phosphoserine residues and result in the formation of dehydroalanine. This product reacts with Cystine and Lysine residues, and new products such as lanthionine and lysinoalanine are formed [34,35], which also hinder protein digestibility by preventing enzymatic proteolysis [36]. Moreover, there is an interchange of disulfide bonds between proteins during the thermal treatment [37], and because of this, some of the resulting non-native complex show resistance to digestion [38]. Similar results were reported in cow milk proteins treated thermal processing [39,40].

Similarly, ultrasounds did not have any major effect on protein digestibility, as their effects were almost similar for all the treated samples. For thermosonicated samples, maximum digestibility was obtained for samples treated for 9 min, followed by samples treated for 3 min and 5 min. A direct relationship exists between the digestibility of proteins and their allergenicity [41,42]. In cow milk, studies reported that ultrasound waves have no effect on the allergenicity of milk samples [43,44]. It can be directly related to the allergenicity, as changes in digestibility do not reflect any changes in allergenicity. Hence, the results obtained are in agreement with those of previous studies conducted on milk samples treated with ultrasound waves.

## 4. Conclusions

Secondary structural changes in milk proteins due to microwave processing, ultrasonication, and thermosonication were evaluated using Fourier Transform Infrared spectroscopy (FTIR). As the applied temperature and time were raised in microwave processing, rearrangement in α-helices occurred, and as a result there was an increase in turns and β-sheets in the proteins. The highest protein digestibility was reported when the milk was treated at 75 °C for 1 min. In the case of ultrasonication, no major changes in secondary structures were observed, except when milk was treated for 9 min, which showed a small rearrangement of α-helix structures. However, no significant changes were observed in the in vitro protein digestibility after ultrasound processing. Very few studies have been conducted on the effect of combined thermal treatment and ultrasound waves, but it is clear that the thermosonicated samples showed poor digestibility, ranging from 68.76 to 78.81%, compared to the microwave samples (72.56–93.4%) and the ultrasound samples (90.20–94.41%), which exhibited higher digestibility.

## Figures and Tables

**Table 1:** Parameters for (a) microwave-processed samples; (b) ultrasound-processed samples, and (c) thermosonicated samples. Note: MW means microwave processing; US means ultrasound processing; TS means Thermosonication processing.

(a) Microwave	Temperature (°C)		
Time (min)	60	75	90
1	MW 60-1	MW 75-1	MW 90-1
3	MW 60-3	MW 75-3	MW 90-3
5	MW 60-5	MW 75-5	MW 90-5
**(b) Ultrasound**		**(c) Thermosonication**	
Time (min)	Samples	Time (min)	Samples
1	US 1	1	TS 1
3	US 3	3	TS 3
5	US 5	5	TS 5
7	US 7	7	TS 7
9	US 9	9	TS 9

**Table 2:** Assignment of secondary structure based on Amide I frequency range [22,23,24,25].

Amide I Frequency (cm^−1^)	Structure
1624–1639	β-sheets
1642–1645	Random coils
1648–1660	α-helix
1662–1697	β-turns

**Table 3:** In vitro protein digestibility of microwave-processed samples (a), ultrasound-processed samples (b), and thermosonicated samples (c). Note: The values are the mean with the standard error. The IVPD (%) of control was 84.42%; small letters indicate the lack of a significant difference at different processing times (*p* > 0.05) based on ANOVA and Tukey’s test.

(a) Microwave	IVPD (%)		
Time (min)	60	75	90
1	74.96 ± 3.08 ab	93.4 ± 4.27 e	78.82 ± 1.52 abcd
3	76.14 ± 4.38 abc	86.06 ± 3.30 cde	79.66 ± 4.65 abcd
5	72.56 ± 1.70 a	73.65 ± 2.62 a	88.63 ± 1.02 de
**(b) Ultrasound**		**(** **c)** **Thermosonication**	
Samples	IVPD (%)	Samples	IVPD (%)
US 1	94.41 ± 2.58 b	TS 1	68.76 ± 4.4 a
US 3	93.05 ± 4.74 b	TS 3	77.47 ± 2.1 abc
US 5	92.48 ± 1.68 b	TS 5	76.84 ± 1.76 abc
US 7	90.20 ± 3.94 b	TS 7	74.25 ± 2.92 ab
US 9	92.86 ± 2.40 b	TS 9	78.81± 1.52 bc

## Data Availability

Data is contained within the article.
